# Enhanced Host Neovascularization of Prevascularized Engineered Muscle Following Transplantation into Immunocompetent versus Immunocompromised Mice

**DOI:** 10.3390/cells8121472

**Published:** 2019-11-20

**Authors:** Luba Perry, Uri Merdler, Maria Elishaev, Shulamit Levenberg

**Affiliations:** 1 Department of Biomedical Engineering, Technion-Israel Institute of Technology, Haifa 32000, Israel; lubashargo@gmail.com (L.P.); urimerdler@gmail.com (U.M.); mikhalchenko89@gmail.com (M.E.); 2 Inter-departmental Program in Biotechnology, Technion-Israel Institute of Technology, Haifa 32000, Israel

**Keywords:** regenerative medicine, skeletal muscle, immunocompetent, immunocompromised, biomaterials, endothelial cells, vascularization, engineered tissue, transplantation

## Abstract

Engineering of functional tissue, by combining either autologous or allogeneic cells with biomaterials, holds promise for the treatment of various diseases and injuries. Prevascularization of the engineered tissue was shown to enhance and improve graft integration and neovascularization post-implantation in immunocompromised mice. However, the neovascularization and integration processes of transplanted engineered tissues have not been widely studied in immunocompetent models. Here, we fabricated a three-dimensional (3D) vascularized murine muscle construct that was transplanted into immunocompetent and immunocompromised mice. Intravital imaging demonstrated enhanced neovascularization in immunocompetent mice compared to immunocompromised mice, 18 days post-implantation, indicating the advantageous effect of an intact immune system on neovascularization. Moreover, construct prevascularization enhanced neovascularization, integration, and myogenesis in both animal models. These findings demonstrate the superiority of implantation into immunocompetent over immunocompromised mice and, therefore, suggest that using autologous cells might be beneficial compared to allogeneic cells and subsequent immunosuppression. Taken together, these observations have the potential to advance the field of regenerative medicine and tissue engineering, ultimately reducing the need for donor organs and tissues.

## 1. Introduction

Contemporary organ and autologous and allogeneic tissue transplantation procedures provide hope for a second chance at life for thousands of patients, who, 60 years ago, would have been considered incurable [1,2]. While organ transplantation is the treatment of choice for end-stage organ failure, the major shortage in available donor organs results in the death of tens of thousands of patients each year [3]. Thus, autograft transplantation has become the gold standard therapy for repairing various large defects in tissues, such as muscle and bone. However, due to size limitations, donor site morbidity, and other complications, a limited degree of success is achieved [4,5,6]. Tissue-engineered autologous grafts designed to replace damaged tissues have emerged as a promising alternative [7]. 

While much progress has been made in the field of tissue engineering, most studies utilize immunocompromised animal models or administer immunosuppressive therapy to study the integration and survival of human engineered tissues in animals [8,9,10,11,12,13,14]. These works have established that in vitro graft prevascularization by human vascular cells is beneficial for graft integration and neovascularization post-implantation [8,9,10,13,15,16,17]. It was also shown that culturing human vascular cells on biodegradable and biocompatible poly (l-lactic acid)/poly (lactide-co-glycolic acid) (PLLA/PLGA) scaffolds combined with fibrin gel results in the spontaneous formation of vessel-like structures in vitro, which later anastomose with the host vasculature upon transplantation into immunocompromised animals [9,16,18]. However, since the immune system plays a major role in the integration and vascularization of transplants within the host [19,20,21,22,23], such immunocompromised models fail to accurately reflect the responses elicited following autologous transplantation and under immunocompetent conditions. Therefore, to better mimic the conditions of autologous transplantation procedures in the clinic, more research is still necessary to elucidate the effect of prevascularization on graft integration and host neovascularization after their transplantation into immunocompetent animals.

As it was shown that angiogenesis and neovascularization are promoted by M2 macrophages and interleukin-17 (IL-17), secreted by activated CD4^+^ T helper cells [24,25,26,27,28], we hypothesized that neovascularization will be improved in immunocompetent compared to immunocompromised animals and that in vitro prevascularization will enhance graft integration and neovascularization after transplantation into immunocompetent animals. Our aim was to utilize intravital imaging to study neovascularization of nonvascularized and prevascularized muscle constructs in immunocompetent and immunocompromised animals. To this end, we constructed a fully murine, vascularized, engineered muscle graft and compared its integration capacities to those of non-prevascularized grafts in immunocompetent versus immunocompromised mice. More specifically, non-prevascularized grafts or grafts seeded with co-cultures of endothelial cells (ECs) and satellite cells (SCs) were transplanted into abdominal wall defects of immunocompromised and immunocompetent mice. Within 18 days of transplantation, grafts transplanted into immunocompetent animals featured more functional vessels as compared to grafts transplanted into immunocompromised control animals. Moreover, prevascularized grafts presented more neovessels and myogenesis and better graft integration in immunocompetent and immunocompromised mice, as compared to their control scaffolds. These results demonstrate the advantages of transplanting prevascularized engineered tissues into immunocompetent organisms and thus suggest that autologous cells might be preferred over allogeneic cells for the construction of engineered tissues.

## 2. Materials and Methods

### 2.1. Cell Culture and Satellite Cell Isolation

Primary mouse fibroblasts and SCs were isolated from the tibialis of 8–10-week-old CD1 mice. Briefly, muscle tissue was separated from bones and cartilage, dissected and minced. Next, muscle segments were placed in 0.25% trypsin-EDTA for enzymatic dissociation (30 min, 37 °C), filtered through a 100 µm membrane (Cell Strainer, BD Falcon) and cultured in rich proliferation medium supplemented with l-Glutamine and gentamycin (BIO-AMF-2, Biological Industries, Ltd., Beit Haemek, Israel). Myogenic cells were separated from fibroblasts using a plating technique which leverages their adherence to gelatin-coated flasks [29]. CD1 mouse lung microvascular endothelial cells were purchased from Creative Bioarray and cultured in endothelial cell medium (ScienceCell), supplemented with 5% fetal bovine serum (FBS) (ScienceCell) and endothelial cell growth supplement (ScienceCell). All incubations were in a 5% CO_2_ humidified atmosphere, at 37 °C.

### 2.2. PLLA/PLGA Scaffold Fabrication

3D porous scaffolds, with pore sizes of 212−600 µm and 93% porosity, composed of PLLA (Polysciences, Warrington, PA, USA) and PLGA (Boehringer Ingelheim, Ingelheim, Germany), were fabricated utilizing a salt-leaching technique, as previously described [8,9,18,30]. Briefly, a 1:1 PLLA: PLGA polymer solution was prepared in chloroform. Next, 0.24 mL of this solution was added to 0.4 g sodium chloride particles in 18 mm-diameter Teflon molds. The chloroform was allowed to evaporate overnight and the scaffolds were washed in distilled water for 8 h. Before each experiment, the 1 mm-thick scaffolds were cut into 6 mm-diameter circles, soaked in 70% (*v/v*) ethyl alcohol for 2 h, and then washed 3 times with PBS.

### 2.3. Multicellular Cultures

1. Co-culture: endothelial cells (0.5/0.8/1 × 10^6^ cells) and satellite cells were cultured at 1:1 ratio.
2. Tri-culture: endothelial cells (0.5/0.8/1 × 10^6^ cells), satellite cells, and fibroblasts were cultured at 5:5:1 ratio.

Satellite cells and fibroblasts were trypsinized at passage 4 and endothelial cells at passage 7 or 8. Cell mixtures prepared at different ratios were resuspended in 4 µL thrombin solution (5 U/mL; Johnson & Johnson Medical, New Brunswick, NJ, USA) before 4 µL fibrinogen (15 mg/mL; Johnson & Johnson Medical, New Brunswick, NJ, USA) were added. Next, each cell suspension was seeded into the PLLA/PLGA scaffolds and allowed to solidify (30 min, 37 °C, 5% CO_2_) inside 6-well, non-tissue culture plates. Culture medium (4 mL) was then added to each well and replaced every other day.

### 2.4. Whole-Mount Immunofluorescence Staining

Whole scaffolds were fixed 14 days post-seeding in 4% paraformaldehyde (PFA; Electron Microscopy Sciences, Hatfield, PA, USA) for 10 min, and subsequently washed with PBS. Cells were permeabilized with 0.3% Triton X-100 (Bio Lab Ltd., Lawrenceville, GA, USA) for 10 min at room temperature (RT), and scaffolds were washed with PBS following a 1-h incubation in blocking serum (10% FBS, 0.1% Triton X-100, 1% glycine) at RT. Next, scaffolds were incubated overnight, at 4 °C, with the following primary antibodies (diluted in blocking solution): rat anti-mouse CD31 (1:50, BD Bioscience, San Jose, CA, USA); rabbit anti mouse Pax 7 (1:100, abcam) and goat anti-desmin (1:50, Santa Cruz Biotechnology, Inc., Santa Cruz, CA, USA). Subsequently, scaffolds were washed four times with PBS and the following secondary antibodies were applied for 3 h: Cy5-conjugated donkey anti-goat IgG (1:100, Jackson Immuno-research Laboratory, West Grove, PA, USA); Cy5-conjugated donkey anti-rabbit IgG (1:100, Jackson Immuno-research Laboratory); Alexa-488-conjugated donkey anti-goat IgG (1:600, Jackson Immuno-research Laboratory); and Alexa-488-conjugated donkey anti-rat IgG (1:400, Jackson Immuno-research Laboratory). Nuclei were counterstained with 4′,6-diamidino-2-phenylindole (DAPI) (1:1000, Sigma-Aldrich). Scaffolds were then washed with PBS and stored in 24-well plates in PBS, at 4 °C, until observation under a Zeiss LSM700 inverted confocal microscope (Carl Zeiss), with 5Χ and 10Χ objective lenses, using ZEN software (Carl Zeiss, Jena, Germany). Further image analysis was conducted using FIJI (Fiji Is Just ImageJ) software.

### 2.5. Vessel Network Eccentricity Determination

Scaffolds bearing co- and tri-cultures stained for CD31 were imaged with a confocal microscope, and eccentricity values of vessel-like structures were calculated using a self-written MATLAB (MATLAB^©^, MathWorks, Natick, MA, USA) algorithm. Briefly, images were transformed into binary images, which were then separated into distinct elements and eccentricity values, ranging from 0 to 1, determined using the “regionprops” function. An eccentricity value of 0 was given for a perfect circle and increased up to 1, as elements became more elongated.

### 2.6. Flow Cytometry

Mouse CD1 endothelial cells were trypsinized and fixed with 4% PFA (20 min, RT), and then rinsed and incubated with rat anti-mouse CD31 antibody (1:50, BD Bioscience, San Jose, CA, USA) for 60 min at RT. Next, cells were thoroughly rinsed and incubated with Alexa 488-conjugated IgG (1:2000; Molecular Probes) for 30 min at 4 °C, and then rinsed and stored in PBS at 4 °C until flow cytometry. Similarly, CD1 satellite cells were fixed and stained with rabbit anti-myosin heavy chain (1:50, Santa Cruz Biotechnology, Inc., Santa Cruz, CA, USA) and goat anti-desmin (1:50, Santa Cruz Biotechnology, Inc., Santa Cruz, CA, USA) antibodies, followed by extensive washing and incubation with the following secondary antibodies: Alexa 488-conjugated IgG (1:2000; Molecular Probes) and Alexa 647-conjugated IgG (1:2000; Molecular Probes). All washes and antibody incubations were performed with PBS supplemented with 0.1% saponin (Sigma-Aldrich) and 10% FBS. Cells were analyzed using the BD LSR-II flow cytometer (Becton Dickenson, San Jose, CA). Data acquisition of 10,000 events per sample was performed without gating. Data were analyzed using FCS Express software (version 5, De Novo Software, Pasadena, CA, USA). A cell sample was also immunolabeled with isotype-matched negative control antibodies and analyzed as a reference.

### 2.7. Engineered Tissue Transplantation

All surgical procedures were conducted according to protocols approved by the Institutional Animal Care and Use Committee of the Technion Israel Institute of Technology. Three types of constructs were tested: empty scaffolds, scaffolds containing SCs only, and scaffolds containing both ECs and SCs (EC+SC). Each construct type (n ≥ 3) was grown for 14 days and then transplanted into two mouse strains: CD1 Flk-1-GFP mice (generously provided by Ondine Cleaver, UT Southwestern) and immunocompromised athymic Nude-Foxn1^nu^ mice (Harlan Laboratories). The 9-week-old mice were anesthetized via intraperitoneal injection of a mixture of ketamine-xylazine (100 mg/kg and 10 mg/kg, respectively), using a 30-gauge needle. Buprenorphine (0.05 mg/kg) was subcutaneously injected 20 min before the procedure and every 12 h thereafter for 3 days. The planned incision site was cleaned with alcohol and iodine to establish an aseptic working field. Then, the abdominal wall was exposed by a ventral skin incision and a 6 mm-diameter full-thickness segment of the rectus abdominis muscle was removed. The engineered construct was sutured in place using 8-0 polypropylene sutures.

### 2.8. Abdominal Imaging Window (AIW)

The AIW was fabricated as previously described [16]. The AIW is made of a reusable stainless-steel ring (17 mm outer diameter, 13 mm inner diameter, and 2.3 mm thick), with a 1.3 mm groove on the side (Figure 2A). Following implantation, the AIW was sealed with a 13 mm circular poly-l-lysine-g-poly (ethylene glycol)-coated glass coverslip. The coverslip was fixed in place by a snap ring (Figure 2A).

### 2.9. Abdominal Imaging Window Implantation

Before use, the AIW was soaked overnight in 70% (*v/v*) ethyl alcohol and washed three times in PBS. The AIW was then implanted, as described by Ritsma et al. [31,32], with some modifications. Briefly, after suturing the engineered tissue in the abdominal wall, a purse-string suture was made through the skin around the incision, using 4-0 silk sutures. Next, cyanoacrylate glue was placed on the interior ring surface of the AIW, which was then fixed in place around the transplanted tissue by applying gentle pressure. The skin was carefully placed in the groove of the AIW. Then, the loops of the purse-string suture were pulled, one by one, to tighten the suture in the groove of the AIW. Lastly, a circular glass coverslip was placed on top and fixed with a snap ring. All mice were monitored closely for 1–2 h to ensure full recovery from the anesthesia. The mice were monitored daily to assess general health, and subsequently assessed by intravital microscopy.

### 2.10. Stabilizing Imaging Device (SID)

A poly(methyl methacrylate) (PMMA) stabilizing imaging device (SID) was manufactured in the department machine shop (Figure S6). Mice were placed inside the SID and stabilized during the intravital imaging session to minimize breathing movements and ensure more focused images.

### 2.11. Intravital Imaging

Intravital microscopy was performed 7, 10, 14, and 18 days post-transplantation with an LSM700 confocal microscope. Blood vessels of immunocompetent CD1 Flk-1-GFP mice were detected via their green fluorescence. To visualize the host vasculature in nude mice, Alexa Fluor^®^ 647-conjugated anti-mouse CD31 antibody (mCD31-X647; Biolegend) (0.5 mg/mL) was intravenously injected into the tail vein and allowed to circulate for ~15 min. Then, the mice were anesthetized (using ketamine-xylazine as described above) and tetramethyl rhodamineisothiocyanate (TRITC)–dextran (10 mg/mL) (average MW 155,000, Sigma–Aldrich) was intravenously injected into the tail vein. Mice were placed in the SID and intravital microscopy was performed. The temperature was maintained at 28 °C, with a heating chamber, throughout the entire imaging session. Mice were euthanized 18 days post-surgery and the grafts were retrieved and fixed in 10% formalin (Sigma-Aldrich).

### 2.12. Vessel Length and Diameter Quantification

Total length (mm) of 10–15 mm-diameter vessels within the constructs 18 days post-transplantation, was calculated by analyzing z-stack confocal projection images, using a self-written MATLAB algorithm. Briefly, vessel network images were converted to binary images using iterative thresholding. Images were then segmented and non-vessel segments were filtered. Filtered images were then skeletonized and segment widths were acquired by dividing segment area by the corresponding skeleton length. Results were then further analyzed using Excel. Two independent in vivo experiments were conducted, with a minimum of three animals per group analyzed in each experiment.

### 2.13. Immunohistochemical Staining

Grafts retrieved 18 days post-transplantation were incubated overnight in a 30% (*w/v*) sucrose solution, embedded in optimal cutting temperature (OCT) compound (Tissue-Tec, San Jose, CA, USA), and frozen for subsequent cryosectioning to 5 μm- and 10 μm-thick sections. For Masson’s trichrome staining, slides were first air-dried and stained with filtered 0.1% Mayer’s hematoxylin (Sigma-Aldrich) for 5 min, followed by distilled water washings and trichrome staining (Sigma-Aldrich) for 2 min. Then, slides were washed twice with 0.2% glacial acetic acid and then with double distilled water. Afterward, slides were dehydrated by serial immersions in increasing concentrations of ethanol and finally dipped in xylene and covered with Vectamount. A Pannoramic MIDI automatic digital slide scanner (3DHISTECH, Budapest, Hungary) was used to image the slides and Pannoramic Viewer software (3DHISTECH, Hungary) was used for the analysis.

### 2.14. Statistical Analysis

Statistical analyses were performed using a computerized statistical program (GraphPad Software, Inc., San Diego, CA, USA). Data are presented as mean ± standard error of the mean (SEM). Data were analyzed by one-way ANOVA, followed by Tukey’s multiple comparison test or by multiple Student’s *t*-tests, where appropriate. *p* values < 0.05 were taken to indicate a statistically significant difference between groups.

## 3. Results

### 3.1. In Vitro Murine Vessel Network Elongation and Murine Satellite Cell Differentiation on PLLA/PLGA Scaffolds

**Figure 1:**
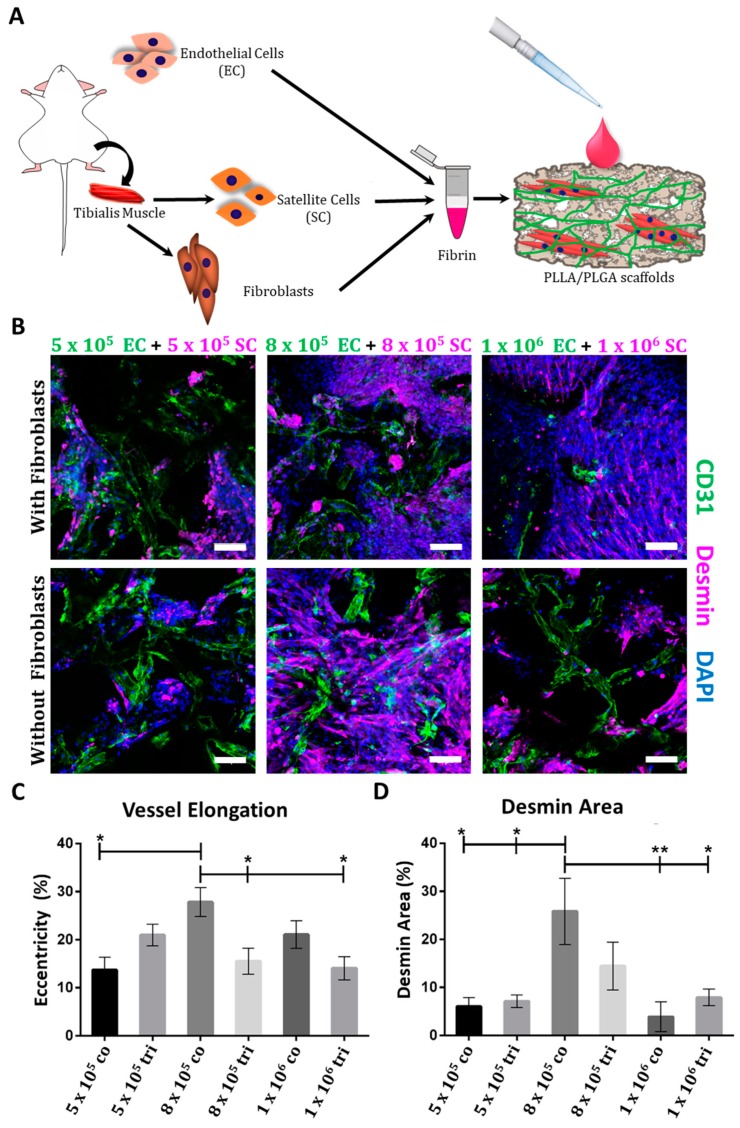
Multicellular culturing strategy. (**A**) A schematic presentation of the cell isolation procedure and the multicellular culture combinations examined. (**B**) Representative confocal images of whole-mount immunofluorescent scaffolds populated with endothelial cells (ECs) and satellite cells (SCs), with or without fibroblasts, 14 days post-seeding. ECs are stained in green, desmin-positive cells are stained in magenta, and nuclei are stained in blue; scale bar = 100 µm. (**C**) Mean percentage (±SEM) of elongated elements with eccentricity scores of 0.95–1 in scaffolds populated with ECs and SCs, with or without fibroblasts, 14 days post-seeding. Data were analyzed by one–way ANOVA, followed by Tukey’s multiple comparison test, n ≥ 4 (* *p* < 0.05). (**D**) Mean (± SEM) desmin expression area in scaffolds populated with ECs and SCs, with or without fibroblasts, 14 days post-seeding. Data were analyzed by one-way ANOVA, followed by Tukey’s multiple comparison test, n ≥ 4 (* *p* < 0.05).

To engineer a fully murine muscle tissue, SCs and fibroblasts were isolated from the tibialis muscle of mice. FACS analysis revealed that 99% of the SCs were desmin-positive (Figure S1) and immunofluorescent staining confirmed that the cells express Pax7, which is a specific SCs marker (Figure S2). After co-culturing the SCs with murine ECs, with or without murine fibroblasts on PLLA/PLGA scaffolds, at various initial densities, for 14 days (Figure 1A), CD31-positive (Figure S3) vessel-like structures were observable (Figure 1B). A significantly higher number of more developed and elongated vessel-like structures was present in co-cultures prepared with 8 × 10^5^ ECs and SCs, as compared to other tested co- and tri-culture groups (Figure 1C). In addition, significantly higher myoblast and multinucleated myotube counts were recorded in these EC-SC co-culture samples, as compared to groups containing other cell ratios (Figure 1B,D, and Figure S4). Therefore, this cell combination and ratio was subsequently used in the animal studies described below.

### 3.2. Neovascularization in Immunocompetent Versus Immunocompromised Mice

**Figure 2:**
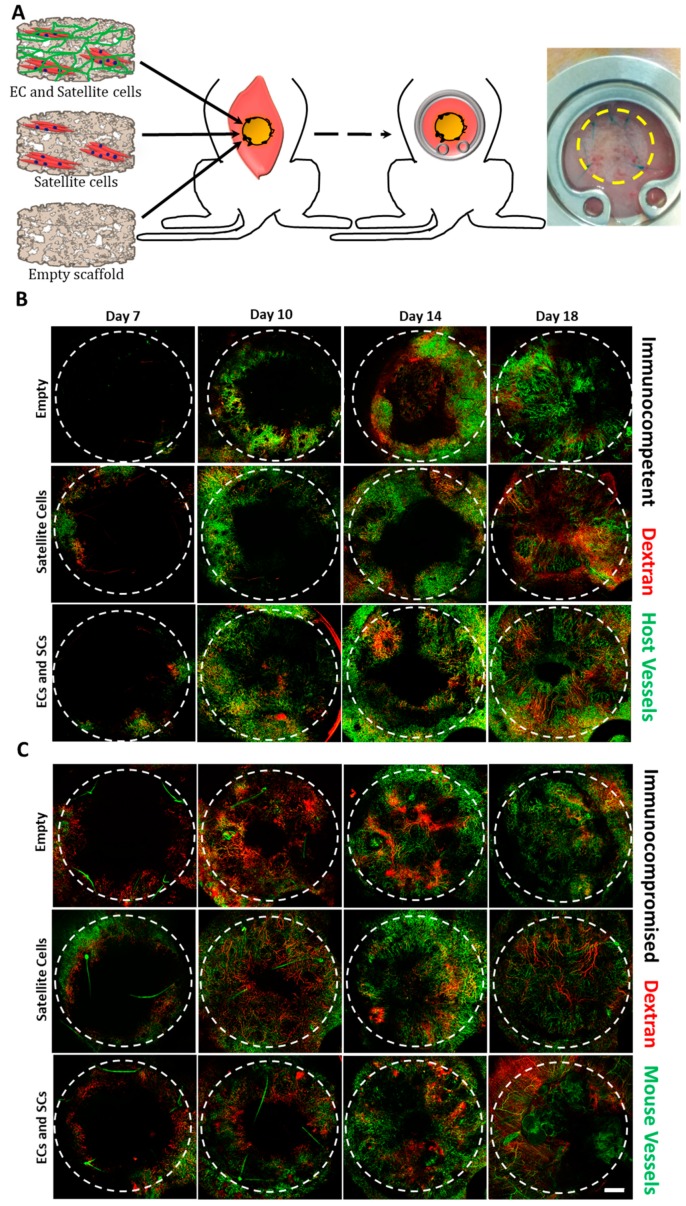
Intravital imaging. (**A**) A schematic presentation of engineered tissue transplantation into defects created in the rectus abdominis muscle of mice, and a representative image of a mouse with an abdominal imaging window (AIW) immediately post-surgery. The area of the transplanted graft is indicated by a yellow dashed circle. Empty, SC-only, and EC-SC-containing scaffolds were grown in vitro for 14 days prior to transplantation. Confocal intravital images were taken 7, 10, 14, and 18 days post-transplantation. Representative intravital confocal images of grafts transplanted into immunocompetent (**B**) or immunocompromised (**C**) mice, as viewed through the AIW. Green: mouse vessels; red: tetramethyl rhodamineisothiocyanate (TRITC)-conjugated dextran. The transplanted graft is indicated by a dashed circle. Scale bar = 1000 µm.

**Figure 3:**
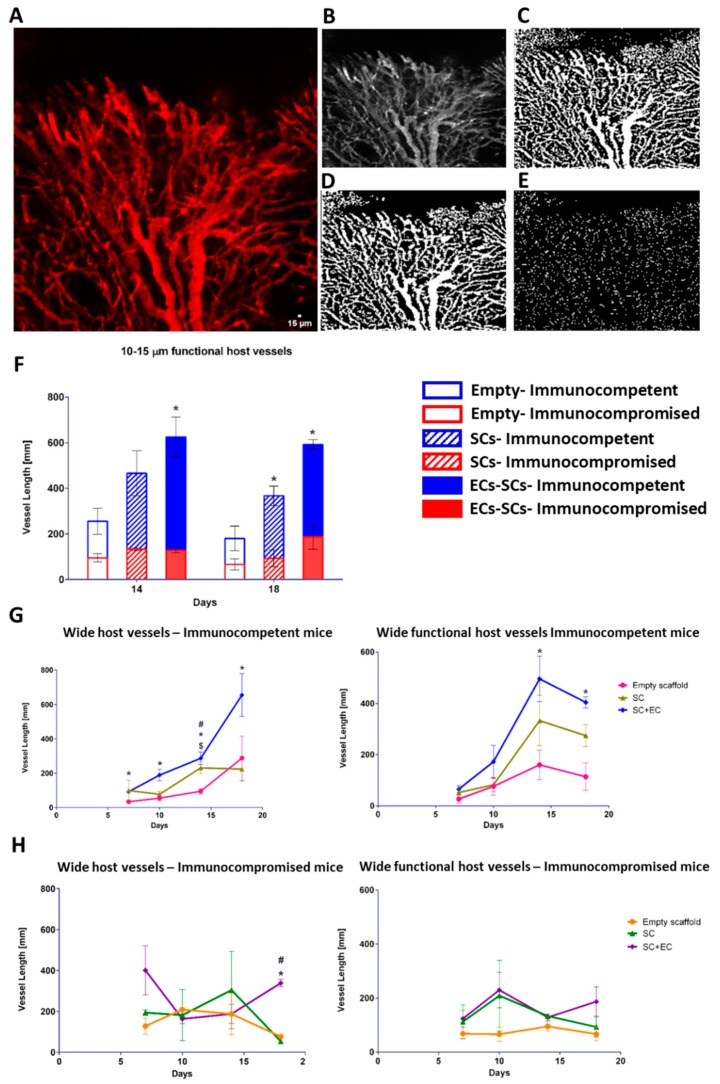
Prevascularization promotes host neovascularization. Empty, SC-only, and EC-SC-containing scaffolds were grown for 14 days in vitro and transplanted into immunocompetent and immunocompromised mice. (**A**) Representative confocal image of host vasculature in the area of the transplanted graft. Scale bar = 15 µm. (**B**) Respective binary image for MATLAB analysis. (**C**) Representative image segmentation. (**D**) Non-vessel segments filtration. (**E**) Skeletonization of filtered image. (**F**) Total vessel length of 10–15 µm-wide functional host vessels penetrating the graft area, on days 14 and 18 post-transplantation. Data are expressed as means ± SEM and were analyzed using the multiple student’s *t*-test, n ≥ 4 (* *p* < 0.05). (**G**) Total vessel length of 10–15 µm-wide host vessels penetrating the graft area on days 7, 10, 14, and 18 after transplantation into immunocompetent mice. Data are expressed as means ± SEM and were analyzed using the multiple student’s *t*-test, n ≥ 4 (* *p* < 0.05 versus empty graft; # *p* < 0.05 versus SC-only graft, $ *p* < 0.05 SC-only graft versus empty graft). (**H**) Total vessel length of 10–15 µm-wide host vessels penetrating the graft area on days 7, 10, 14, and 18 after transplantation into immunocompromised mice. Data are expressed as means ± SEM and were analyzed using the multiple student’s *t*-test, n ≥ 4 (* *p* < 0.05 versus empty graft; # *p* < 0.05 versus SC-only graft).

After a 14-day in vitro incubation period, scaffolds were transplanted into an abdominal defect in immunocompetent and immunocompromised mice (Figure 2A). To visualize functional blood vessels throw, the AIW, TRITC-dextran was injected into immunocompetent mice, which express Flk-1 GFP blood vessels. Figure S5A demonstrates the presence of dextran in functional blood vessels. Host vasculature was visualized penetrating the graft area throughout the 18-day post-transplantation period (Figure 2). Majority of the host vessels were positive for both Flk-1 and CD31 (Figure S5B). By day 18, the entire area of the graft in both immunocompetent and immunocompromised models was completely vascularized by host vessels (Figure 2). An approximate 5-fold and 3-fold increase in the total vessel length of wide functional host vessels penetrating the EC-SC-bearing scaffolds was observed in immunocompetent versus immunocompromised mice on days 14 and 18, respectively (Figure 3A–F). In addition, at all tested time points, the total vessel length of wide host vessels penetrating the EC-SC-bearing scaffolds in immunocompetent animals was significantly higher compared to vessels penetrating empty scaffolds (Figure 3G). This paralleled, 3-fold increase in the total vessel length of functional vessel in the grafts of the immunocompetent group on day 14 and day 18 post-transplantation compared to empty scaffolds (Figure 3G); SC-only scaffolds contained a similar total vessel length of functional host vessels as did empty scaffolds (Figure 3G). On day 18, the total vessel length in EC+SC scaffolds implanted into immunocompromised was approximately 6-fold greater than in SC-only and empty scaffolds (Figure 3H).

### 3.3. Graft Integration and Myogenesis in Immunocompetent and Immunocompromised Mice

**Figure 4:**
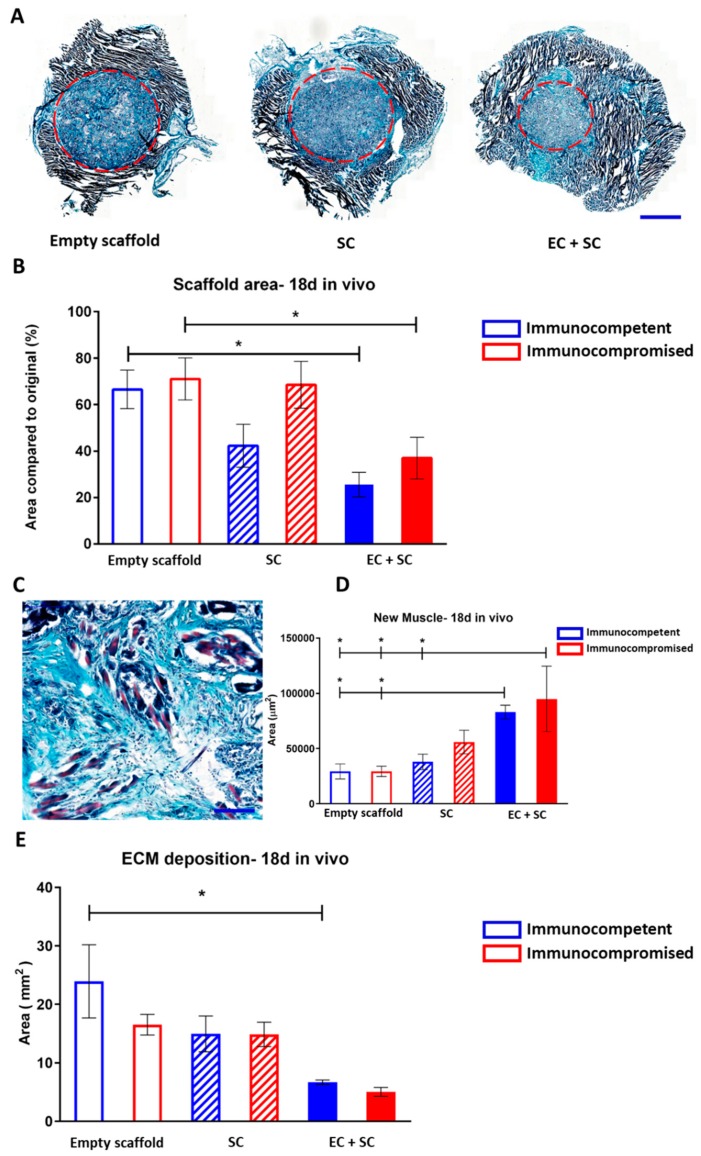
Graft integration and new muscle formation in vivo. (**A**) Representative images of Masson’s trichrome-stained grafts, 18 days post-transplantation into immunocompromised animals. The final graft area is indicated by a red dashed circle. Scale bar = 2000 µm. (**B**) Final versus initial scaffold area quantification. Data are presented as mean area ± SEM and were analyzed by one-way ANOVA, followed by Tukey’s multiple comparison test, n ≥ 4 (* *p* < 0.05). (**C**) Representative large-magnification image of a Masson’s trichrome-stained EC+SC graft, 18 days post-transplantation. Newly formed muscle bundles are seen in light purple. (**D**) Quantification of the area of new myofibers. Data are expressed as mean area ± SEM and were analyzed by one-way ANOVA, followed by Tukey’s multiple comparison test, n ≥ 4 (* *p* < 0.05). (**E**) Extracellular matrix (ECM) deposition area quantification. Data are presented as mean area ± SEM and were analyzed by one-way ANOVA, followed by Tukey’s multiple comparison test, n ≥ 4 (* *p* < 0.05).

Grafts were retrieved 18 days post-surgery and were measured (Figure 4A,B) and analyzed for signs of myogenesis (Figure 4C,D). In both models, the retrieved EC+SC grafts demonstrated seamless integration with the host tissue and were surrounded by native muscle (Figure 4A). Empty scaffold grafts explanted from both immunocompromised and immunocompetent mice were mostly un-replaced by the host tissue (Figure 4B). In sharp contrast, approximately 70% of EC+SC scaffolds, in both mouse models, had been replaced by host muscle tissue (Figure 4B). In parallel, in both immunocompromised and immunocompetent mice, the grafts featured approximately 3-fold more new muscle bundle coverage as compared to empty scaffolds (Figure 4C,D), indicating that EC presence in the graft accelerated myogenesis in scaffolds implanted into muscle tissue. In line with these observations, extracellular matrix (ECM) deposition quantification revealed that most of the empty scaffold grafts were composed of ECM (Figure 4E). In immunocompetent animals, EC+SC grafts featured almost 4-fold less deposited ECM compared to empty scaffolds, indicating that the tissue regeneration process, in these grafts, was dominated by muscle formation rather than ECM deposition (Figure 4E).

## 4. Discussion

Allotransplantations and autotransplantations are the gold standard treatment for repair of damaged tissues and failed organs [1,2]. However, the major shortage in donor organs and significant donor site morbidity demand exploration of alternative solutions [3]. Organ decellularization and recellularization, 3D printing, and scaffold-mediated cell self-assembly are some of the approaches assessed in recent years for their potential in addressing functional organ replacement demands [10,33,34,35,36,37]. In such methodologies, the utilized cells can be autologous or allogeneic combined with administration of immunosuppressive drugs to avoid graft rejection. Due to the many complications and side effects associated with immunosuppression [28], autologous cells are a preferable cell choice. However, most studies assessing transplanted tissue engraftment and neovascularization utilized immunocompromised animals or immunosuppression and hence do not accurately mimic autologous tissue transplantation. However, since the immune system plays a critical role in transplant integration [19,20,21,22], the integration and neovascularization processes are bound to differ in immunocompetent animals versus immunocompromised ones. Therefore, there remains a need to establish a model to understand tissue engraftment and neovascularization processes in immunocompetent animals.

It was previously shown that anti-inflammatory M2 macrophages promote angiogenesis [24,25,27,38] and that the pro-inflammatory interleukin-17 (IL-17) cytokine, primarily produced by activated CD4^+^ T helper cells, promotes angiogenesis and neovascularization [26,39,40]. Following muscle injury, neutrophils are the first responders, followed by M1 macrophages which peak around day 2. Next, a transition between M1 phenotype and M2 phenotype occurs, which is a key event for the normal progression of muscle regeneration and neovascularization [27,41]. CD4+ T cells play a major role in this transition. In a different muscle injury model of periphery artery disease, Kwee et al. showed recently that conditioned media of Th2 and Th17 T-cells, delivered from an injectable alginate biomaterial into the ischemic hindlimb muscle of mice, enhanced angiogenesis and myogenesis 14 days post-delivery [42]. Therefore, we hypothesized that post-transplantation neovascularization will be enhanced in immunocompetent animals bearing all of the natural immune components compared to athymic nude immunocompromised mice, which lack T cells. Here, a 3D, prevascularized murine muscle was constructed using fibrin gel and PLLA/PLGA scaffolds and then transplanted into immunocompetent mice and compared to those transplanted into immunocompromised mice. Vascularization, myogenesis, and integration were monitored for 18 days through an AIW.

An inherent vasculature, such as a capillary bed that can readily connect to the host vascular system, is essential to ensure implantability of thick and viable tissue [43,44,45]. The presence of ECs in the implants is important not only for anastomosis with the host vasculature but also for secretion of angiogenic factors that induce host invasion into the graft. A previous study conducted by Koffler et al. showed that 3 weeks post-implantation into abdominal wall defect, human ECs were almost completely replaced by mouse ECs and this was accompanied by a reduction in human capillary morphogenesis genes while angiogenic factors like VEGF and FGF-2 were still highly expressed [15]. In our previous studies, we used human ECs and human fibroblasts or smooth muscle cells to construct a vessel-like network within the engineered tissue that were transplanted into immunocompromised animals [8,9,15,16,18]. In the current study design, in order to transplant the engineered tissue into immunocompetent animals, we used murine ECs, SCs, and fibroblasts. The addition of stromal cells to the culture is crucial to support ECs in the formation of stable blood vessels. Here, vessel-like networks also formed in EC+SC co-culture without the addition of fibroblasts (Figure 1), likely due to a small fibroblast population that proliferated post-isolation. As indicated from the nuclei staining in Figure 1, many cells did not express desmin and since post-isolation only 99% of the cells were desmin-positive (Figure S1), it is most likely that there were also fibroblasts in the culture that proliferated and supported the ECs in their vessel-like network formation.

We have previously reported on successful transplantation of engineered vascularized muscle tissues, constructed on PLLA/PLGA scaffolds, into an abdominal wall musculature defect in immunocompromised mice [9,15,16,18,46]. Synthetic biodegradable PLLA/PLGA scaffolds, made from polymers approved for clinical use, were shown to promote vessel formation in vitro and to enable host vessel penetration in vivo [9,13,15,18]. When integrating HUVEC and human fibroblast co-cultures into the scaffolds, Lesman et al. [18] and Koffler et al. [15] found that prevascularization of 3D engineered muscle tissue in the laboratory promoted host vessel penetration post-transplantation and improved muscle functionality in vivo. Similarly, we have previously [9] described transplantation of a 3D vascularized muscle, composed of ECs derived from elderly patients, smooth muscle cells (SMCs), and human myoblasts seeded on PLLA/PLGA scaffolds. The vascularized muscle tissue integrated and anastomosed with the host vasculature within 9 days of transplantation into the abdominal wall of nude mice. In our recent work [16], we demonstrated that genetically engineered human skeletal muscle, which secreted angiopoietin 1 and vascular endothelial growth factor, promoted myogenesis and neovascularization of the transplant. In our previous studies, we successfully demonstrated the use of vascular cells, which have clinical applicability and can be easily isolated from elderly patients for the construction of autologous tissues. Yet, while highly promising with respect to clinical applicability of the construct, the models involved immunocompromised animals. In line with our hypothesis, we found that by 14 and 18 days post-transplantation, total length of wide functional host vessels was significantly higher in immunocompetent animals as compared to those in the immunocompromised animals (Figure 3), suggesting faster and more effective neovascularization of the transplant when the host immune system is intact and fully functional. Moreover, at all assessed post-transplantation time points, total vessel length observed in the prevascularized muscle tissue in immunocompetent mice was significantly higher compared to non-prevascularized control scaffolds. In contrast, in the immunocompromised mice, significant differences between the scaffold types were only observed on day 18 (Figure 3). Yet, when focusing on the 10–15 µm-wide functional host vessels, their total vessel length in immunocompetent mice was significantly higher on both days 14 and 18 post-transplantation, in scaffolds bearing ECs as compared to empty scaffolds (Figure 3). These observations suggest that prevascularization of the transplant promotes host neovascularization in both immunocompetent and immunocompromised models. They also clearly indicate that neovascularization is enhanced in immunocompetent mice compared to immunocompromised animal models. Total vessel length of SCs bearing scaffolds was significantly higher compared to empty scaffolds on day 14 post-implantation into immunocompetent animals (Figure 3G). This correlates with previous findings demonstrating secretion of pro-angiogenic factors from differentiating myogenic cells that stimulate vascularization of the surrounding tissue [47]. We have also demonstrated in the past that by adding mice myoblasts, a significant increase in functional vessel density was observed compared to ECs/Fibroblasts grafts [8]. Incorporation of ECs in the transplanted muscle enhanced graft integration and new muscle bundle formation in both immunocompromised and immunocompetent mice (Figure 4). No significant difference in myogenesis was observed between immunocompetent and immunocompromised animals (Figure 4D). Muscle regeneration has shown to be regulated by interactions between the immune system and skeletal muscle. Following acute muscle injury, the skeletal muscle responds with a Th1 cytokine-driven innate immune response, which in turn stimulates SC activation, proliferation, and subsequently, myogenesis. Th1 cytokines are produced by either T-helper cells or macrophages. It was demonstrated that the presence of M1 macrophages and neutrophils at the site of the injured muscle is most crucial for the onset of myogenesis [41]. As both models tested here possessed normal levels of both macrophages and neutrophils, the lack of significant difference in the myogenesis process is, therefore, not surprising. These results strongly support the benefits of transplantation into immunocompetent animals, thus advocating the use of autologous cells over allogeneic cells for the construction of engineered tissues for transplantation to avoid immunosuppression. Moreover, we demonstrated that tissue prevascularization is beneficial not only in immunocompromised models, as previously shown, but also in immunocompetent models. Other allogeneic cell sources are constantly being explored due to the challenges involved in autologous cell isolation and proliferation. Yet, as our results clearly indicate enhancement of tissue neovascularization, which is crucial for thick tissue survival post-transplantation upon transplantation into immunocompetent animals, more strategies for successful isolation and proliferation of autologous cells should be explored. Moreover, additional work is still needed to elucidate transplantation sequelae in immunocompetent models before we can construct an autologous, clinically relevant engineered tissue, to overcome the massive shortage in donor organs for transplantation.

## 5. Conclusions

In summary, we describe here a three-dimensional, vascularized murine muscle construct that was transplanted into immunocompetent mice and immunocompromised animals, as a control. We compared neovascularization, integration, and myogenesis of the engineered muscle tissues in both transplantation models. Utilizing intravital imaging, we demonstrated that graft neovascularization was significantly enhanced in immunocompetent compared to immunocompromised mice. We also demonstrated promotion of host neovascularization and enhanced myogenesis and graft integration in prevascularized constructs, compared to the non-prevascularized constructs, in both models. Taken together, our findings strongly support the supremacy of in vitro prevascularization as well as transplantation into immunocompetent animals to enhance graft neovascularization. These observations suggest that using autologous cells for the construction of engineered tissues will be beneficial over allogeneic cells in terms of implant neovascularization, which is crucial for successful engraftment.

## Supplementary Materials

The following are available online at https://www.mdpi.com/2073-4409/8/12/1472/s1, Figure S1. Flow cytometry analysis of desmin- and myosin-positive mouse SCs. Figure S2. Representative confocal images of SCs, 7 days post-seeding. Figure S3. Flow cytometry analysis of CD31-positive mouse ECs. Figure S4. Representative confocal images of whole-mount immunofluorescent scaffold populated with ECs and SCs, 14 days post-seeding. Figure S5. Representative intravital images, obtained through the AIW, of an EC- and SC-embedded graft 18 days post-transplantation. Figure S6. Image of a mouse stabilized in a custom-made stabilizing imaging device (SID) for intravital confocal imaging.
