# Supplementary material for: Enhanced Host Neovascularization of Prevascularized Engineered Muscle Following Transplantation into Immunocompetent versus Immunocompromised Mice
Source: Cells. 2019 Nov 20;8(12):1472. doi: 10.3390/cells8121472 (PMC6953003; doi:10.3390/cells8121472)
Supplement: Supplementary file 1 [file cells-08-01472-s001.pdf]

A

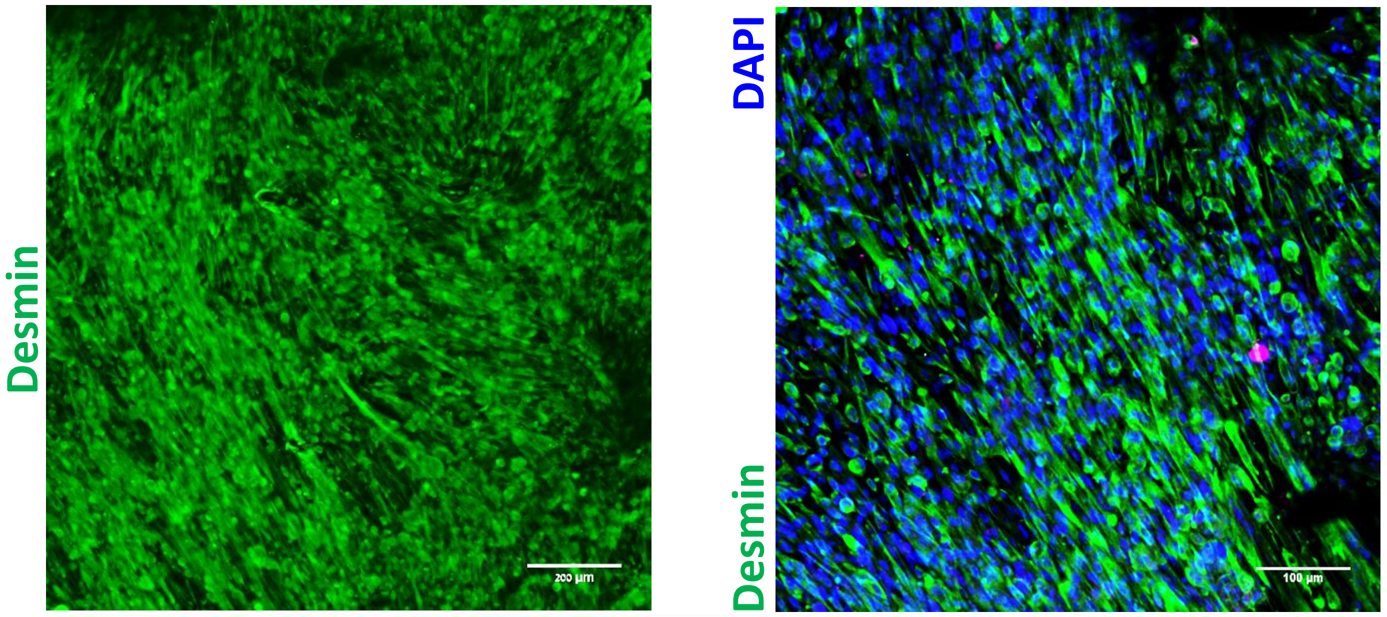

B

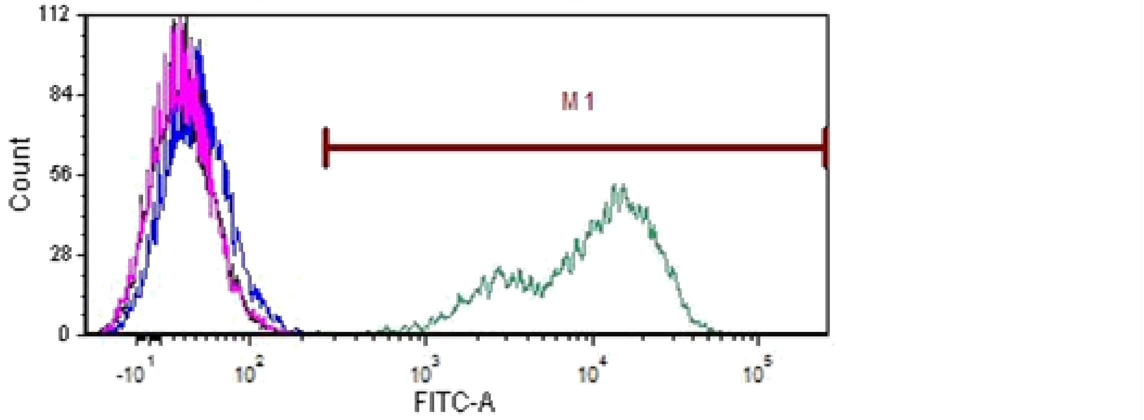

Histogram #	Filename	Parameter	Low bound	High bound	# of Events	% of gated cells	Median	Geometric Mean	CV
1	SCs-2nd antibody only	FITC-A	267.95	254300.03	0	0.00	n/a	n/a	n/a
2	Unstained SCs	FITC-A	267.95	254300.03	2	0.02	283.388	283.53	7.02
3	SCs-desmin (A488)	FITC-A	267.95	254300.03	9180	99.43	10410.50	8389.41	80.03
4	SCs-MYH (A647)	FITC-A	267.95	254300.03	7	0.08	312.08	344.72	26.87

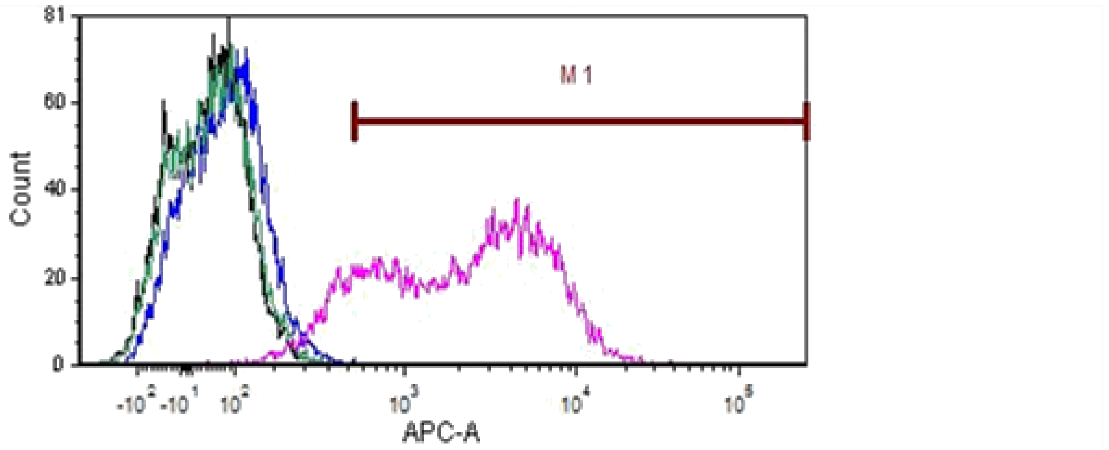

Histogram #	Filename	Parameter	Low bound	High bound	# of Events	% of gated cells	Median	Geometric Mean	CV
1	SCs-2nd antibody only	APC-A	542.12	262144.00	8	0.09	773.55	936.94	78.47
2	Unstained SCs	APC-A	542.12	262144.00	2	0.02	648	645.89	11.39
3	SCs-desmin (A488)	APC-A	542.12	262144.00	12	0.13	847.8	1011.04	79.5
4	SCs-MYH (A647)	APC-A	542.12	262144.00	7922	85.18	2907.9	2683.90	115.68

Figure S1. Flow cytometry analysis of desmin- and myosin-positive mouse SCs. (A) Representative confocal images of SCs, 7 days post-seeding. Desmin-positive cells are stained in green and nuclei are stained in blue. **(B)** Approximately 99% of the SCs isolated from the tibialis were desmin-positive. **(C)** Approximately 85% of the cells within the cell culture were myosin-positive.

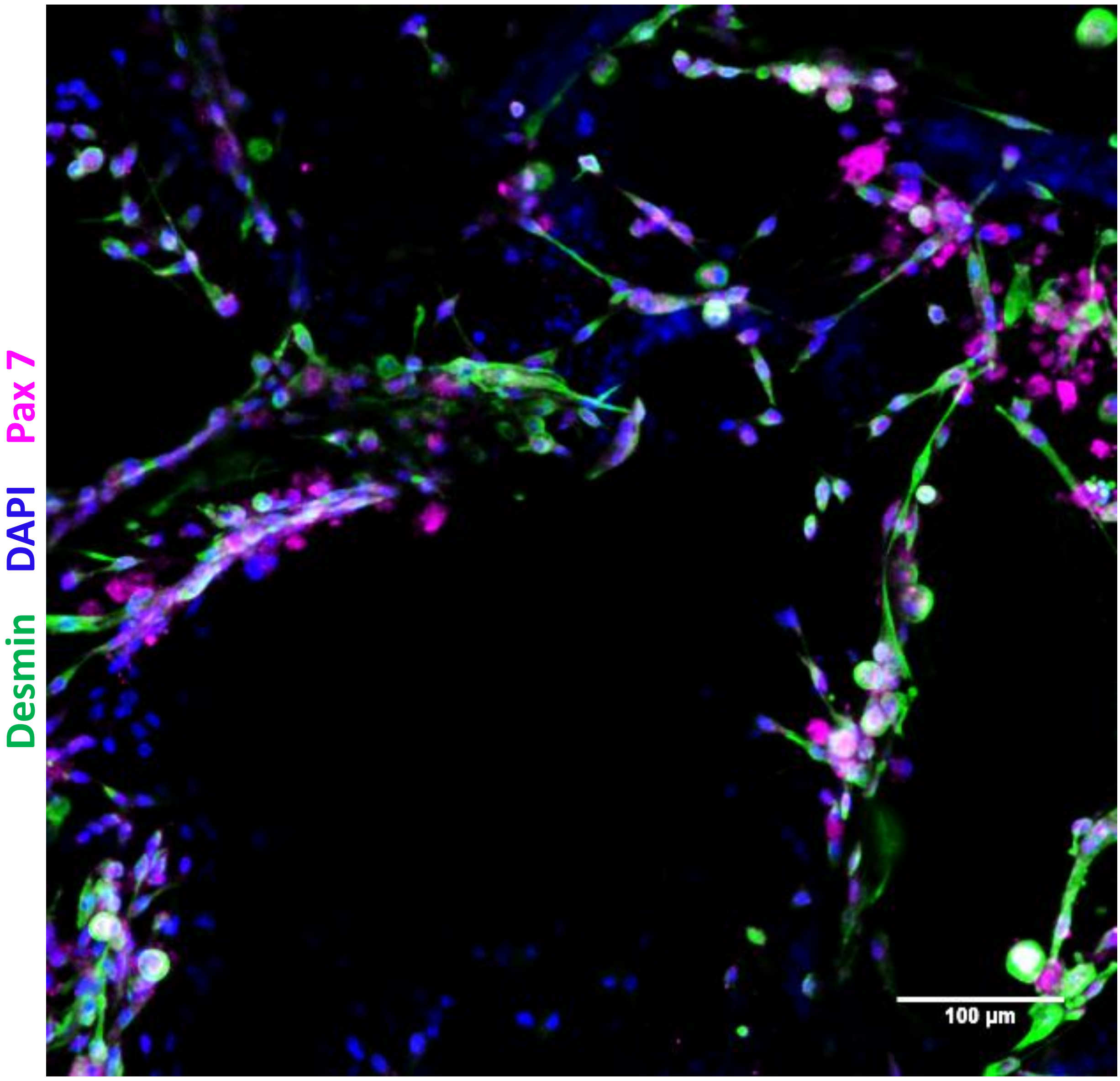

Figure S2. Representative confocal images of SCs, 7 days post-seeding. Desmin-positive cells are stained in green, Pax 7 positive cells are stained in magenta and nuclei are stained in blue; scale bar = 100 µm.

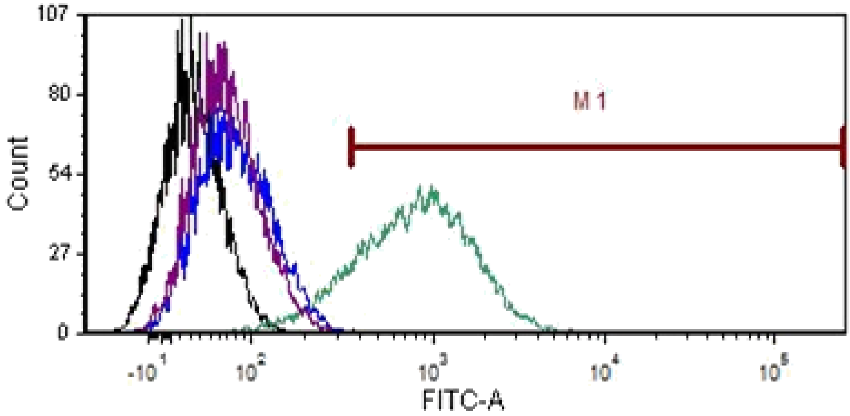

Histogram #	Filename	Parameter	Low bound	High bound	# of Events	% of gated cells	Median	Geometric Mean	CV
1	Unstained ECs	FITC-A	356.74	254300.77	0	0.00	n/a	n/a	n/a
2	ECs-CD31 (A488)	FITC-A	356.74	254300.77	7265	84.09	915.56	945.31	68.07
3	ECs-2nd antibody only	FITC-A	356.74	254300.77	7	0.08	391.98	839.68	193.35
4	SCs-CD31 (A488)	FITC-A	356.74	254300.77	5	0.06	382.11	623.12	177.31

Figure S3. Flow cytometry analysis of CD31-positive mouse ECs. Approximately 84% of the mouse ECs were CD31-positive.

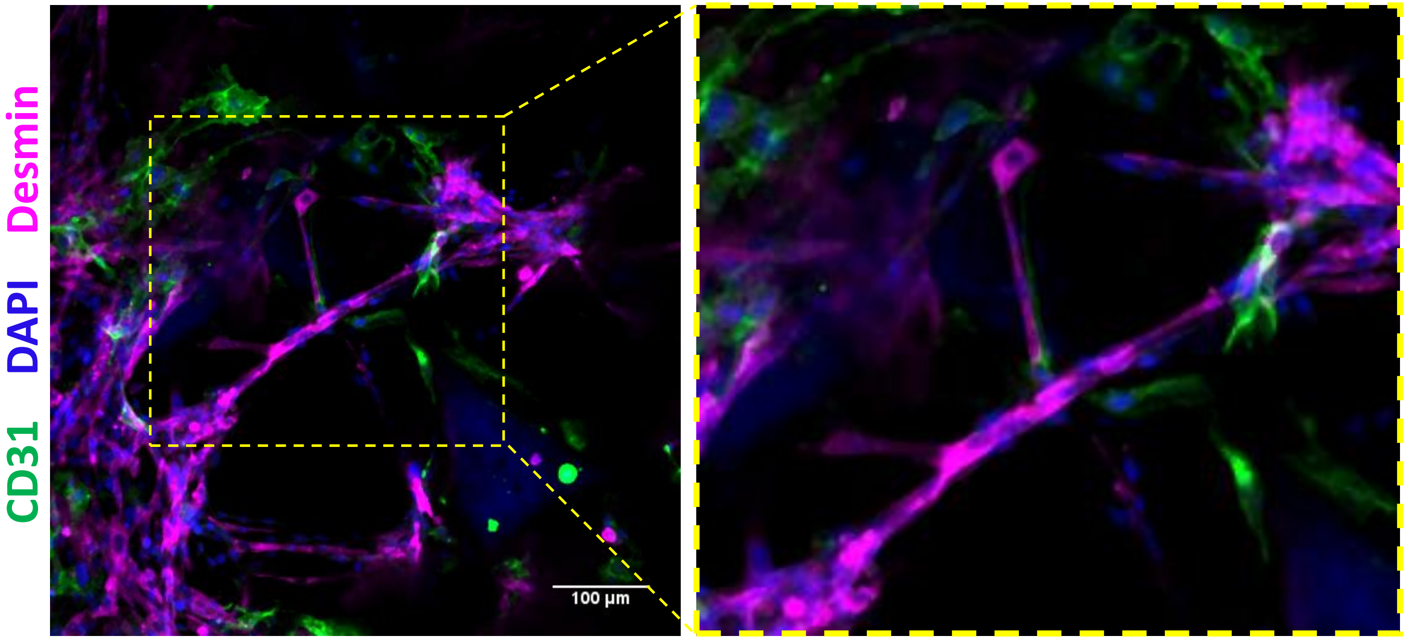

Figure S4. Representative confocal images of whole-mount immunofluorescent scaffold populated with ECs and SCs, 14 days post-seeding. ECs are stained in green, desmin-positive cells are stained in magenta and nuclei are stained in blue; scale bar = 100 µm.

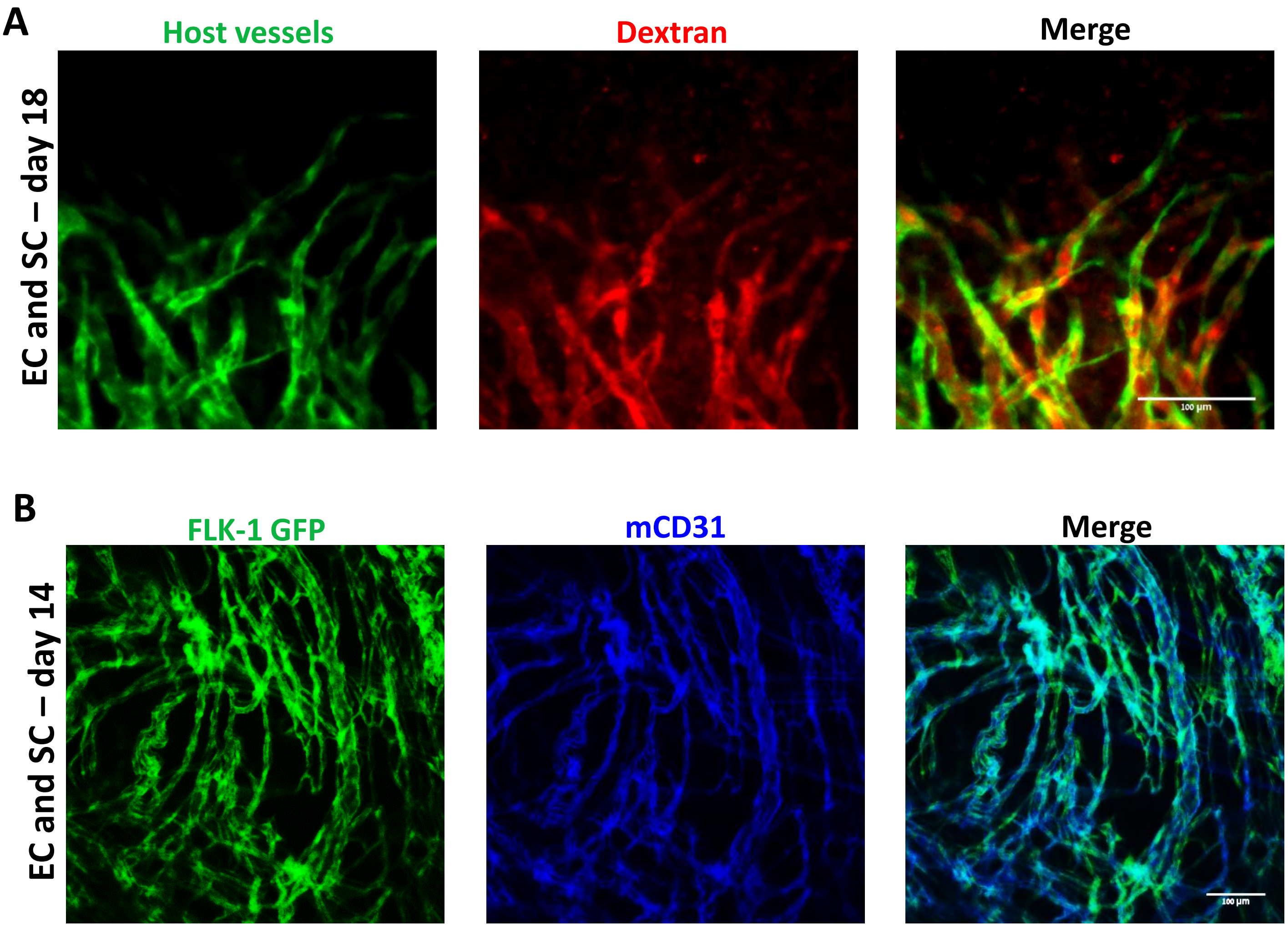

Figure S5. (A) Representative intravital images, obtained through the AIW, of an EC- and SC-embedded graft, 18 days post-transplantation. Green: host vasculature; red: TRITC-conjugated dextran. Scale bar = 100 µm. **(B)** Representative intravital images, obtained through the AIW, of an EC- and SC-containing graft, 14 days post-transplantation. Green: Flk-1 GFP vessels; blue: mouse CD31. Scale bar = 100 µm.

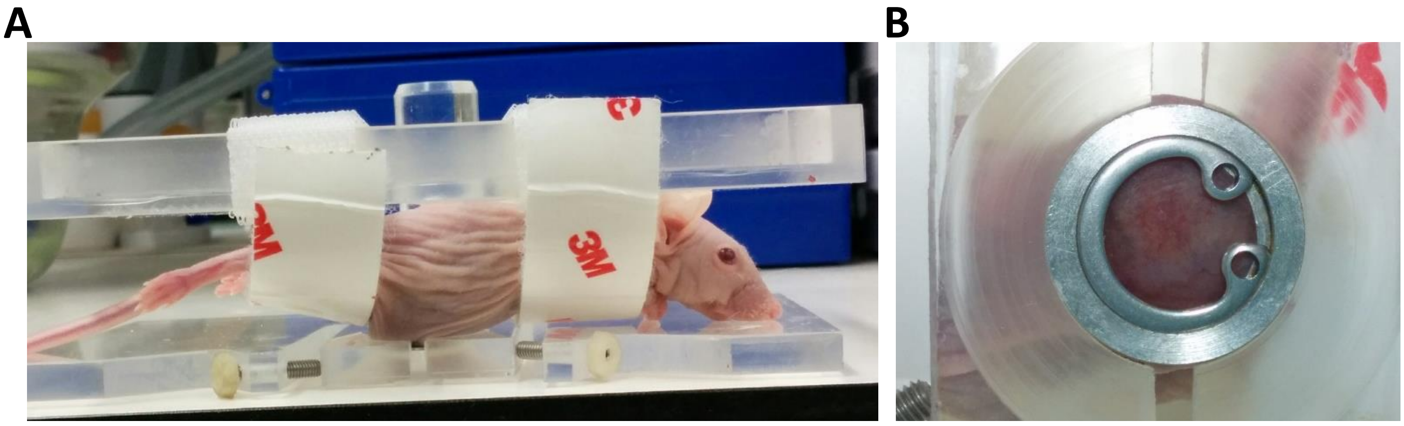

Figure S6. Image of a mouse stabilized in a custom-made SID for intravital confocal imaging. (A) lateral view of an anesthetized mouse in the SID. **(B)** Representative image of a mouse with an abdominal imaging window (AIW) 14 days post-surgery.
